# All-optical charging and charge transport in quantum dots

**DOI:** 10.1038/s41598-020-71601-x

**Published:** 2020-09-10

**Authors:** Jacob Hastrup, Lorenzo Leandro, Nika Akopian

**Affiliations:** DTU Department of Photonics Engineering, Technical University of Denmark, 2800 Kgs. Lyngby, Denmark

**Keywords:** Optics and photonics, Optical techniques, Quantum information

## Abstract

Optically active quantum dots are one of the promising candidates for fundamental building blocks in quantum technology. Many practical applications would comprise of multiple coupled quantum dots, each of which must be individually chargeable. However, the most advanced demonstrations are limited to devices with only a single dot, and individual charging has neither been demonstrated nor proposed for an array of optically active quantum dots. Here we propose and numerically demonstrate a method for controlled charging of multiple quantum dots and charge transport between the dots. We show that our method can be implemented in realistic structures with fidelities greater than 99.9%. The scheme is based on all-optical resonant manipulation of charges in an array of quantum dots formed by a type-II band alignment, such as crystal-phase quantum dots in nanowires. Our work opens new practical avenues for realizations of advanced quantum photonic devices, for instance, solid-state quantum registers with a photonic interface.

## Introduction

Optically active quantum dots (QDs), as opposed to gate-defined QDs, provide a spin-photon interface^1–4^ which can act as a fundamental unit in a quantum computer^5^ or quantum network^6^, onto which quantum information can be stored, manipulated and read-out. In particular, the spin states of a confined electron or hole in a QD make up a qubit which can be efficiently manipulated optically^7,8^. Many steps towards the realization of a QD-based spin-photonic quantum network have already been demonstrated for isolated QDs. These include picosecond optical coherent single spin initialization^8–11^, manipulation^7,12^ and read-out^13,14^, spin-photon entanglement^2,3^, photon-to-spin teleportation^15^ and distant spin–spin entanglement^16^. However, to exploit the full potential of such a quantum network, multi-qubit nodes consisting of several coupled QDs in an array are necessary. Such a QD array would constitute a quantum register in which advanced quantum operations, such as CNOT-gates^17^, can be performed, enabling universal quantum computing and communication of multi-qubit states.

The fabrication of semiconductor QDs is now reaching the point where multi-QD structures can be engineered and reliably realized with atomic precision in nanowires thanks to recent progress on crystal phase control during the growth^18^. However, the fabrication alone does not enable it as a spin-photonic interface as the spin-carrying charges first have to be individually loaded into the QDs in a controlled manner. Such charge loading can be reliably achieved in single QD systems^19^. However, a scalable method allowing controlled site-specific charging of an array of optically active QDs remains yet to be proposed.

**Figure 1:**
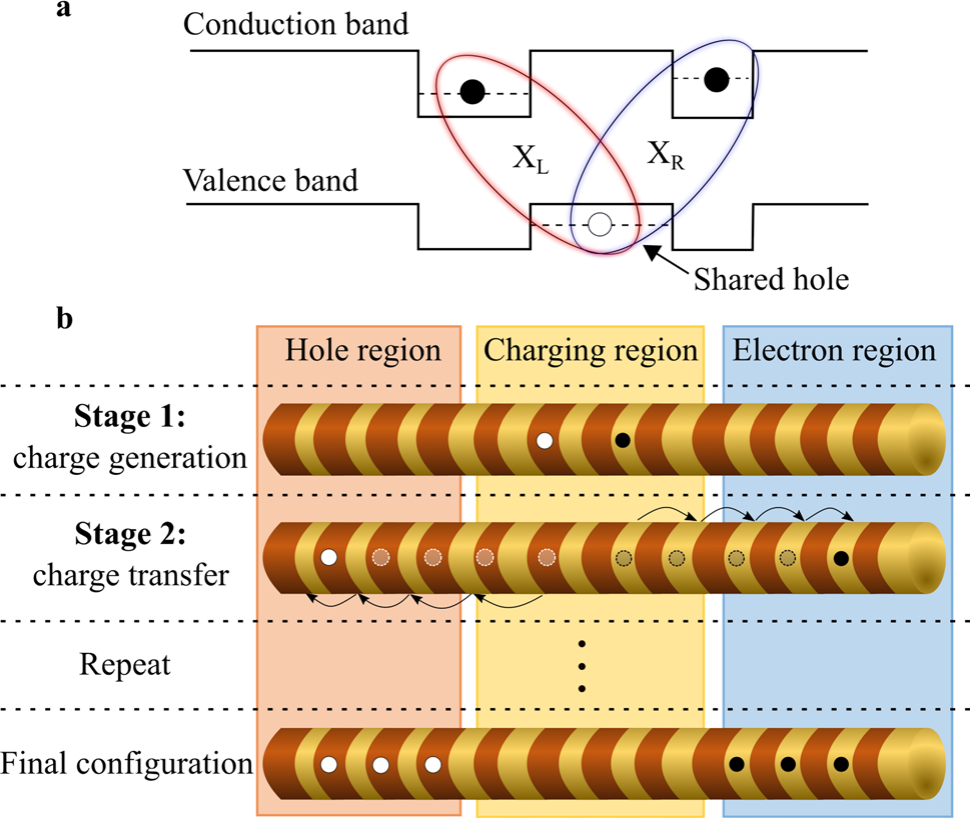
All-optical charge initialization concept in a type-II QD array. (**a**) Schematics of two spatially indirect excitons, X_L_ and X_R_, sharing a common hole in adjacent QDs formed by a type-II band alignment. Black circles represent electrons and white circles represent holes. (**b**) Two-stage process of charge initialization in an array of QDs. First, an electron–hole pair is generated in a long-lived configuration in a “Charging region”. Secondly, the charges are transferred along the QD array, electrons to the “Electron region” and holes to the “Hole region”. These two stages are repeated to initialize multiple charges within the structure.

Here, we present a novel method for controlled all-optical charging of multiple QDs exploiting the unique advantages of an array of QDs formed by a type-II band alignment. Such an array can be reliably achieved using crystal-phase quantum structures in nanowires^18,20–26^ in, for example, InP or GaAs. In these structures, excited electrons and holes are confined within different spatial regions, as shown in Fig. 1a. Consequently, excitons are spatially indirect and can share a single charge state. A hole can, therefore, act as a link, connecting spatially separated electron states, which we exploit in this work (likewise, an electron can connect two spatially separated hole states). Even though the hole and electron of an exciton in a type-II bandstructure are spatially separated^21,27^, the exciton oscillator strength can still be substantial in an array of type-II QDs^24,28^, allowing for efficient optical exciton generation.

The structure we are considering is an array of type-II QDs in a nanowire, as illustrated in Fig. 1b. Our scheme uses two sequential stages: In Stage 1, charge pairs are generated in a “Charging region” and in Stage 2 single charges are transferred to opposite regions of the nanowire, labeled as the “Electron region” and “Hole region” in Fig. 1b. These two stages are repeated to obtain the desired charge configuration. If only a single type of charge is required, e.g. electrons, one can add a reservoir in one end of the nanowire where the unused charges, e.g. the holes, are dumped.

**Figure 2:**
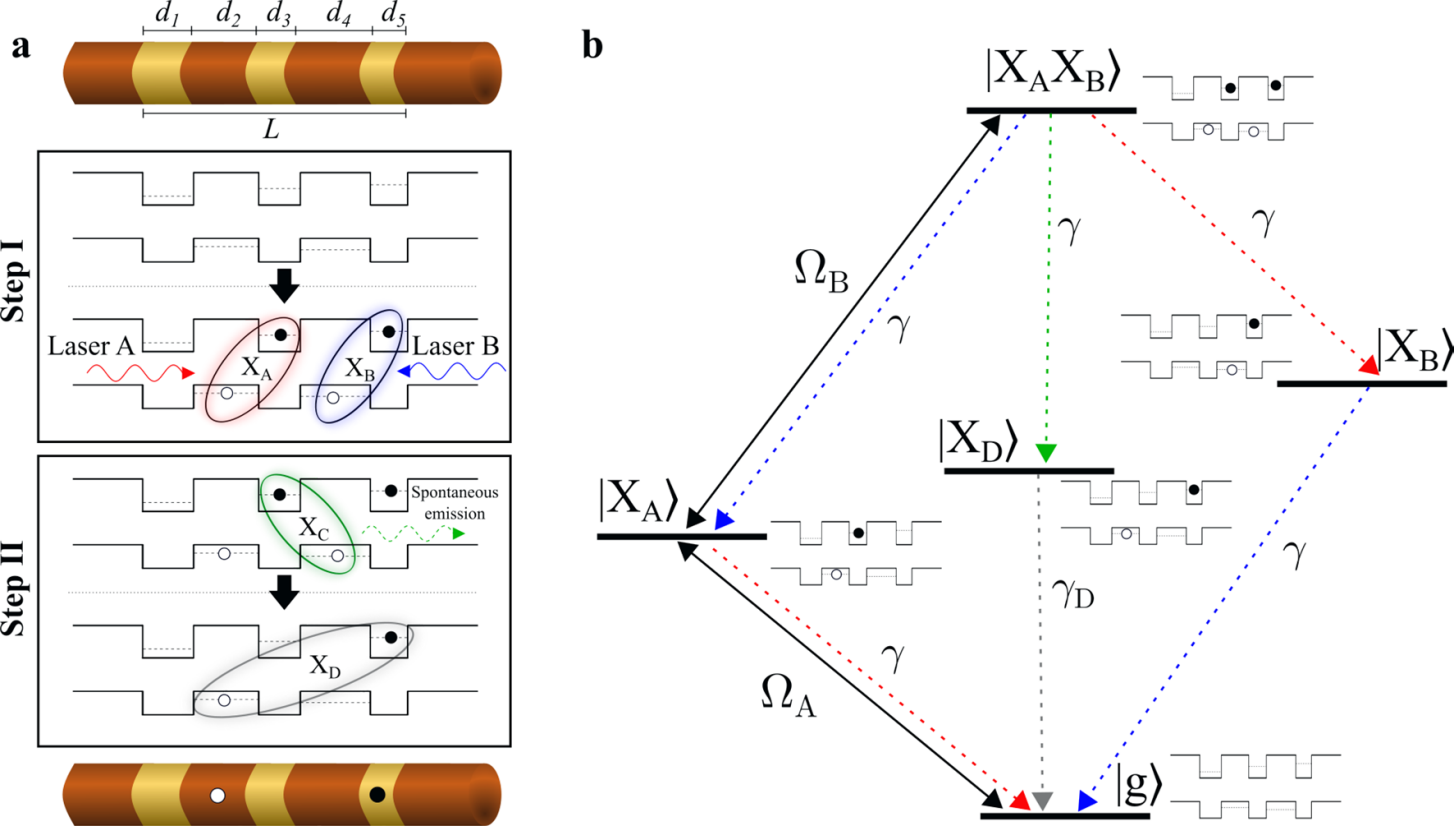
Charge generation in type-II QDs. (**a**) Step I: Two lasers, A and B, simultaneously resonantly excite two neighboring excitons, X_A_ and X_B_. Step II: The electron of exciton X_A_ spontaneously recombines with the hole of exciton X_B_, leaving behind a spatially separated electron–hole pair forming a long-lived exciton, X_D_. (**b**) Energy level diagram of the five states involved in the charge generation scheme. The dashed arrows indicate spontaneous emission paths with rates denoted by $\gamma$’s. The solid double arrows indicate the two driving lasers with Rabi frequencies ${\Omega }_{A}$ and ${\Omega }_{B}$, respectively. Colors represent the relative energies of the transitions, i.e. blue corresponds to the highest energy, red to the lowest and green is intermediate. Coulomb interactions modify the energy level of one exciton depending on the presence of another. Thus, if laser A is resonant on transition $\left. {\left| g \right.} \right\rangle \leftrightarrow \left. {\left| {X_{A} } \right.} \right\rangle$ laser B should thus be resonant to transition $\left. {\left| {X_{A} } \right.} \right\rangle \leftrightarrow \left. {\left| {X_{A} X_{B} } \right.} \right\rangle$ and not $\left. {\left| g \right.} \right\rangle \leftrightarrow \left. {\left| {X_{B} } \right.} \right\rangle$ (see Supplementary Section S2).

The charge generation in Stage 1 is realized using the two steps illustrated in Fig. 2a:

- Step I: The initial state is the ground state in which no charges are excited in the structure. Two lasers then simultaneously resonantly excite two neighboring excitons, labeled X_A_ and X_B_. The structure is designed such that X_A_ and X_B_ have different energies to allow for their individual optical addressing.
- Step II: The electron of X_A_ and the hole of X_B_ now form a third exciton, labeled X_C_, which can also spontaneously recombine. Spontaneous recombination of X_C_ leaves behind a long-lived exciton, X_D_.

The large spatial separation of the charges of X_D_ results in a considerably lower electron–hole overlap and so this final charge configuration can be thought of as stable, with a lifetime multiple orders of magnitude larger compared to an electron–hole pair in adjacent QDs (like X_A_, X_B_, X_C_), as shown in the Supplementary Information Section S1. X_D_ is the final configuration of Stage 1 in Fig. 1b, where we have generated one electron and one hole in separate regions of the structure. The spin state can subsequently be initialized using well-known spin pumping techniques^8–11^.

**Figure 3:**
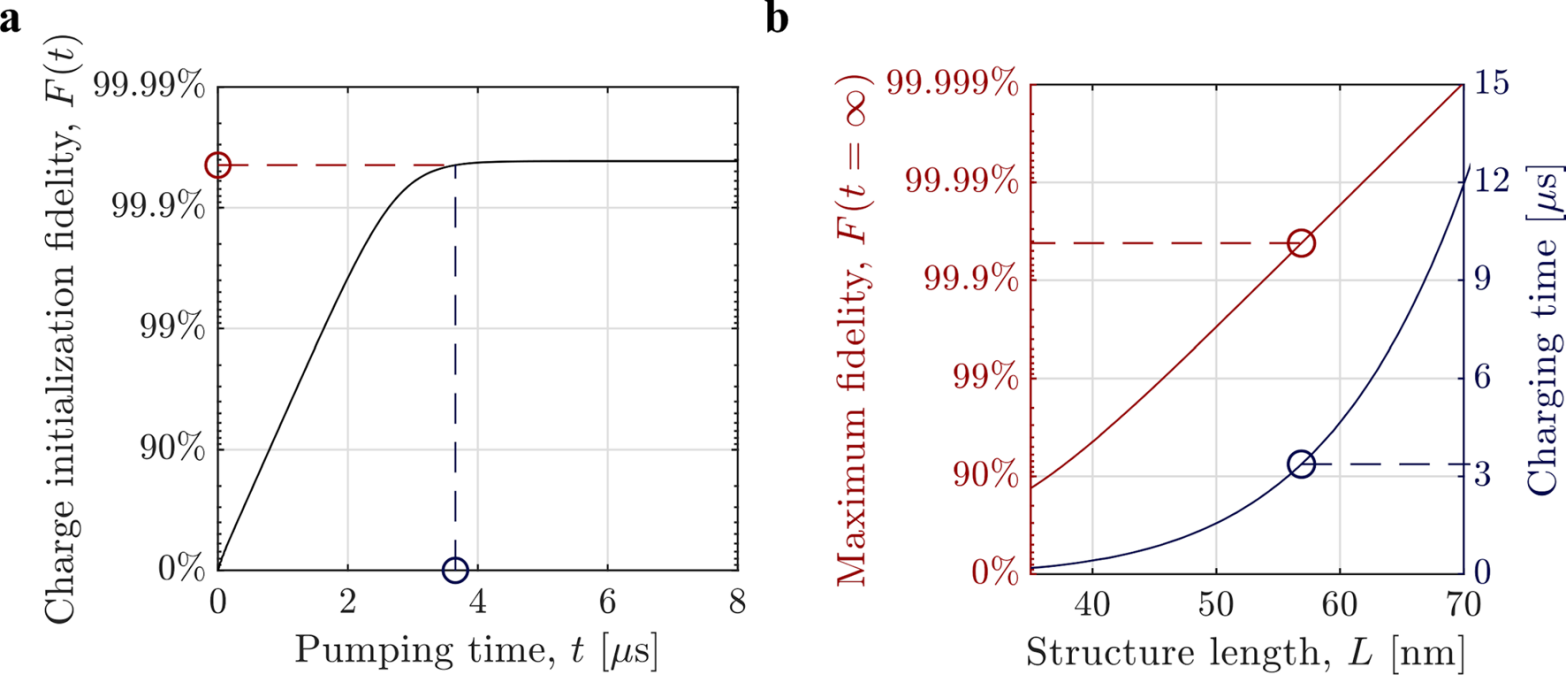
Dynamics of charge generation. (**a**) Time evolution of the charging fidelity, calculated using the Master equation for an InP nanowire with an array of crystal-phase QDs of dimensions $\left[{d}_{1},{d}_{2},{d}_{3},{d}_{4},{d}_{5}\right]=\left[11\;\mathrm{nm},14\;\mathrm{nm},9\;\mathrm{nm},15\;\mathrm{nm},8\;\mathrm{nm}\right]$. The dashed lines indicate the threshold set to define the pumping time. (**b**) Maximum fidelity (red) and charging time (blue) as a function of total structure length, $L={d}_{1}+{d}_{2}+{d}_{3}+{d}_{4}+{d}_{5}$. The circles and dashed lines mark the values for $L=57\;\mathrm{nm}$, corresponding to the structure used in (**a**). The laser Rabi frequencies in both plots are ${\Omega }_{A}={\Omega }_{B}=20\gamma$, ensuring the condition $\Omega \gg \gamma$.

We now calculate the preparation fidelity of X_D_. In principle, the presence of X_D_ can be heralded by the detection of the photon spontaneously emitted by X_C_. However, with a low collection and detection efficiency, this heralding method will be inefficient. A more efficient method would be to track the resonance fluorescence from the recombination of X_A_ or X_B_. Once exciton X_C_ recombines, the lasers can no longer excite X_A_ and X_B_ and thus resonance fluorescence from these excitons are quenched, heralding the preparation of exciton X_D_. Such dynamical charge sensitivity was recently experimentally demonstrated^29^. This method is further explained in Supplementary Section S5. Alternatively, one can just pump the QDs with the two lasers (A and B) for a sufficient time, since once the system reaches the end of Step II the lasers will have no further effect as they are resonant only with their respective exciton energies. A detailed energy diagram of the involved states in our scheme is shown in Fig. 2b. A fundamental limit to the success probability of this approach is the spontaneous recombination rate of exciton X_D_, denoted ${\gamma }_{D},$ arising from a non-zero electron–hole overlap. Due to the similar sizes of the QDs, we assume the recombination rates of excitons X_A_, X_B_, and X_C_ to be equal and denoted $\gamma ,$ and much quicker than the recombination of X_D_, i.e. $\gamma \gg {\gamma }_{D}$ (these assumptions are justified in Supplementary Section S1). The steady-state fidelity, *F*, i.e. the probability to observe only exciton X_D_ after sufficiently long pumping time, can be calculated using the Master equation as (see Supplementary Section S3):

1 $$F(t=\infty )\approx 1-4 \; ({\gamma }_{D}/\gamma )$$

where we have assumed sufficient laser powers such that ${\Omega }_{A}/\gamma ,{\Omega }_{B}/\gamma \gg 1$. This assumption ensures that both excitons are generated before the first exciton recombines and is experimentally feasible (see Supplementary Section S3). Equation (1) shows that the fidelity approaches unity as the recombination rate of exciton X_D_, ${\gamma }_{D}$, decreases, i.e. when ${\gamma }_{D}/\gamma \to 0$, as expected. As an example, for a realistic nanowire QD array, we numerically calculate ${\gamma }_{D}/\gamma =1{0}^{-4}$ resulting in $F=99.96\%$. The dimensions of the QDs should be chosen to make each transition individually addressable via appropriate laser frequencies and to suppress tunneling effects. Figure 3a shows the fidelity as a function of pumping time. We see that the fidelity reaches its maximum after $t \approx 4\;{\upmu }{\text{s}}$, as indicated by the dashed lines. Note that the charging time cannot be decreased by increasing the laser powers, as the limiting time factor is the spontaneous recombination rate of exciton X_C_. This charging time is relatively long compared to typical qubit coherence times of QD defined qubits, but we emphasize that the purpose of the charge generation protocol is to place the charges in the desired positions, before any subsequent quantum operations, requiring qubit coherence, are carried out. Yet, if ultra-fast charging is required, one can stimulate the recombination of exciton X_C_ with a third laser in pulsed mode, enabling charging on a ps timescale.

In Fig. 3b we show how the maximum fidelity and charging time depends on the size of the structure. In these calculations, the structure length is varied while keeping the relative QD sizes constant. By increasing the size, both $\gamma$ and ${\gamma }_{D}$ are decreased with ${\gamma }_{D}$ decreasing more quickly such that the ratio ${\gamma }_{D}/\gamma$ decreases (see Supplementary Section S1), yielding a larger fidelity. For example, to increase the fidelity from $F=99.9\%$ to $F=99.99\%$ requires an increase of the structure size by about 14%, from $L=54\;\mathrm{nm}$ to $L=62\;\mathrm{nm}$. The trade-off is an increased pumping time required to reach steady-state, ~ 2.4 times longer in this case. Thus by making the structure sufficiently large, very high fidelities can be reached with a pumping time on the order of μs. A proposal for a practical experimental scheme to implement and verify the generation of long-lived charges using two tunable lasers is given in the Supplementary Section S5. We note that the calculations of ${\gamma }_{D}/\gamma$ above were made in the absence of Coulomb interactions, pure dephasing, and phonon-assisted processes. The effect of Coulomb interactions will be to increase $\gamma$ compared to ${\gamma }_{D}$, and therefore our calculations are, in fact, underestimating the charging fidelity (see Supplementary Section S2). Pure dephasing has the effect of slightly reducing the fidelity while increasing the required pumping time, due to less efficient pumping of the system (see Supplementary Section S3). Phonon interactions might limit the fidelity due to phonon-assisted pumping of transition $\left. {\left| {X_{D} } \right.} \right\rangle \leftrightarrow \left. {\left| {X_{A} } \right.} \right\rangle \left. {\left| {X_{B} } \right.} \right\rangle$. Therefore the energy levels should be engineered to minimize this effect.

**Figure 4:**
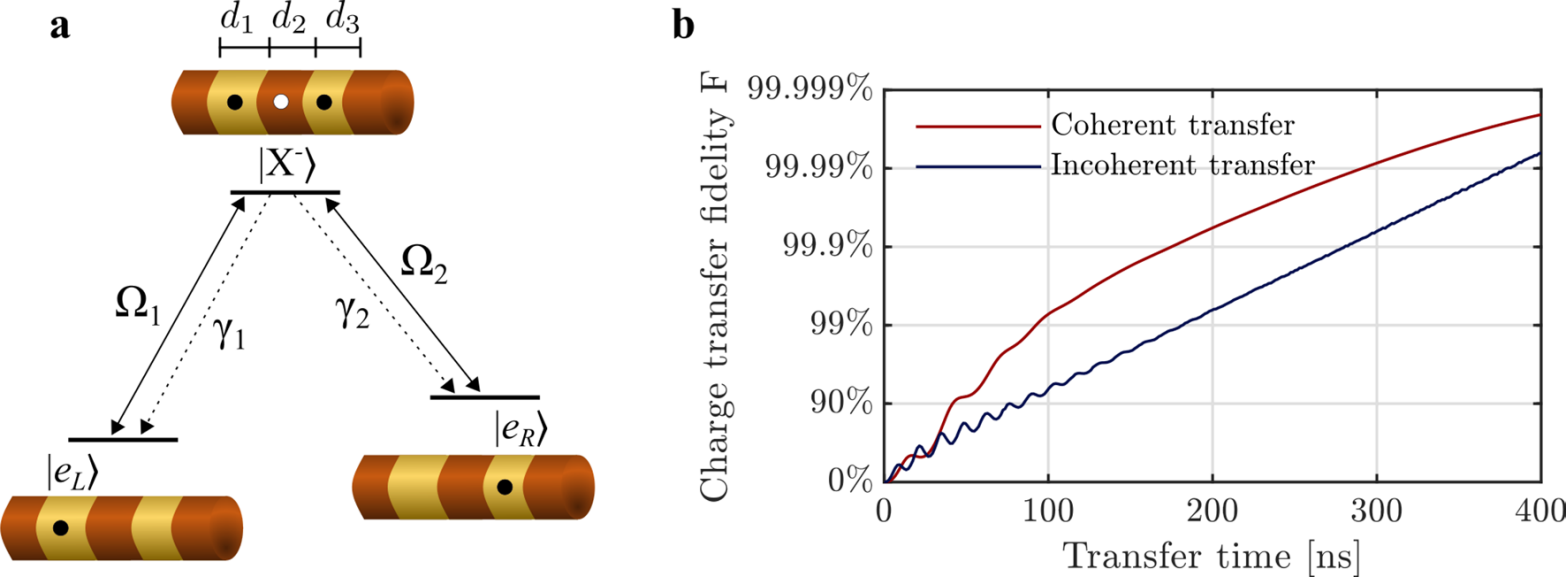
Coherent and incoherent charge transfer. (**a**) $\Lambda$-type system formed by two location states of an electron, $|{e}_{L}\rangle$ and $|{e}_{R}\rangle$, and a spatially distributed negatively charged exciton state, $|{X}^{-}\rangle$. Driving transition $|{e}_{L}\rangle \leftrightarrow |{X}^{-}\rangle$ with a single resonant pump, ${\Omega }_{1}\ne 0$, ${\Omega }_{2}=0$, incoherently pumps the electron to state $|{e}_{R}\rangle$ due to the spontaneous recombination of the left electron with the hole, $|{X}^{-}\rangle \to |{e}_{R}\rangle$. Coherent charge transfer can be obtained, e.g. using the STIRAP scheme, when both transitions are driven, ${\Omega }_{1}\ne 0$, ${\Omega }_{2}\ne 0$ in a suitable pulse sequence (see Supplementary Section S4). (**b**) Fidelities of coherent and incoherent transfers plotted against transfer times, for a structure of dimension $[{d}_{1},{d}_{2}, {d}_{3}] =[10\;\mathrm{nm},10\;\mathrm{nm},7\;\mathrm{nm}]$ for which ${1/\gamma }_{1}=44\;\mathrm{ns}$ and ${1/\gamma }_{2}=22\;\mathrm{ns}.$

Once a pair of charges has been generated, they can be subsequently transferred to their desired positions, as illustrated in Stage 2 of Fig. 1b. This charge transfer process is described in Fig. 4. A single electron can be moved from one location state, $|{e}_{L}\rangle$, to another, $|{e}_{R}\rangle$, via an intermediate spatially distributed negatively charged exciton, $|{X}^{-}\rangle$ (see Fig. 4a). These three states form a $\Lambda$-type system, where the ground states are the spatially separated electron states $|{e}_{L}\rangle$ and $|{e}_{R}\rangle$. This system allows optical transfer of an electron between the QDs. The easiest way to realize such transfer is to resonantly pump the transition $|{e}_{L}\rangle \leftrightarrow |{X}^{-}\rangle$ with a single laser, i.e. ${\Omega }_{1}\ne 0$, ${\Omega }_{2}=0$ in Fig. 4a. This corresponds to the generation of an exciton consisting of the middle hole and the right electron, in the presence of the left electron. Spontaneous recombination of the left electron with the hole, i.e. the transition $|{X}^{-}\rangle$ → $|{e}_{R}\rangle$, eventually leaves the electron in the right QD. Once the electron is in state $|{e}_{R}\rangle$ the laser pump has no further effect, and so by pumping for a sufficiently long time, the electron is effectively transferred from one location to another. This is analogous to well-known optical spin-pumping techniques^9^, but with location-states instead of spin-states. This pumping technique is incoherent, i.e. any quantum coherence of the electron is lost, which, however, is fine for initializing the positions of the charges. Instead, if one wishes to extend the scheme for a more general transport of qubits, coherent transfer is required. Such a coherent transfer can be realized in our system using e.g. the stimulated Raman adiabatic passage (STIRAP) scheme^30^ as proposed in literature^17^. In Fig. 4b we calculate and compare the fidelities of coherent and incoherent transfers against transfer times, expressed as the duration of the involved laser pulses (see Supplementary Section S4 for details). We see that for laser powers of $\Omega =20{\gamma }_{1}$ (the same power used in the charge generation stage), the performances of the two schemes are comparable, with the STIRAP scheme resulting in larger fidelities for the considered pulse-lengths. For realistic pulse intensities, we can obtain high fidelity coherent transfer within 0.1 μs, i.e. faster than coherence times in state-of-the-art systems utilizing, for instance, spin-echo techniques^31^. With the STIRAP protocol the charge can thus be moved several steps before spin coherence is lost. Generally, high laser powers make coherent charge transfer the fastest option, as incoherent transfer is limited by the spontaneous recombination rate of the charged exciton (Supplementary Section S4). On the other hand, incoherent charge transfer is a much easier option, requiring only a single laser, and no accurate control of pulse timing and duration.

To conclude, we have presented a novel all-optical scheme for a scalable method for charging an array of QDs. In addition, we have shown how to transfer charges in such an array of QDs, both coherently and incoherently. The utilization of spatially indirect excitons for coupling of spatially separated charge states, is central for our scheme. The structures modeled in our work can be readily fabricated with current growth technology of crystal-phase switching in nanowires. Along with well-known methods for optical preparation, manipulation, and read-out of the spin states, our method enables the realization of advanced multi-qubit structures with a photonic interface such as, for instance, a scalable all-optically controlled QD-based quantum register, not possible with existing schemes.

## Supplementary information

Supplementary Information.

## Data Availability

The data supporting the findings of this study are available from the corresponding author on reasonable request.
