# Supplementary material for: All-optical charging and charge transport in quantum dots
Source: Sci Rep. 2020 Sep 10;10:14911. doi: 10.1038/s41598-020-71601-x (PMC7483522; doi:10.1038/s41598-020-71601-x)
Supplement: Supplementary file 1 — Supplementary Information. [file 41598_2020_71601_MOESM1_ESM.pdf]

Supplementary Information

All-optical Charging and Charge Transport in Quantum Dots

Jacob Hastrup, Lorenzo Leandro, and Nika Akopian*

DTU Department of Photonics Engineering, Technical University of Denmark, 2800 Kgs. Lyngby, Denmark
**e-mail: nikaak@fotonik.dtu.dk*

S1. Calculation of spontaneous recombination rates

For our calculations, we consider quantum dots (QDs) formed by wurtzite and zincblende crystal phases of an InP nanowire, with material parameters as shown in table S1. The spontaneous recombination rate of an exciton in a QD in a homogeneous medium is given by[1]

$$\gamma = d^2\omega^3 n / (3\pi\varepsilon_0\hbar c^3)$$

where $\hbar\omega$ is the energy of the exciton, n is refractive index of the medium and d^2 is the transition dipole moment. In the following, we consider the strong confinement regime where confinement energies dominate over Coulomb interactions, such that the exciton wavefunction can be factorized into an electron and a hole wavefunction, $\psi_e(r)$ and $\psi_h(r)$ respectively. This regime is valid for small QDs, such as those studied in our work. The effects of Coulomb interactions are discussed in section S2. The dipole moment is then given by[2]:

$$d^2 = e^2|M|^2/(m_0{}^2\omega^2)|\langle\psi_h|\psi_e\rangle|^2 \quad ,$$

where $|\langle\psi_h|\psi_e\rangle|^2 = \int dr\psi_h(r)^*\psi_e(r)$ is the envelope function overlap, e is the electron charge, m_0 is the free electron mass and $|M|^2$ is the momentum matrix element between the conduction band and the valence band. To calculate γ we therefore only need to calculate

$|\langle\psi_h|\psi_e\rangle|^2$. Neglecting Coulomb interactions, the envelope wavefunctions are given by the solutions to the Schrödinger equation:

$$[-\hbar^2/(2m_i)\nabla^2 + V_i(r)]\,\psi(r) = E\,\psi(r) \quad ,$$

where $i \in [e, h]$ denotes electrons or holes, m_i is the effective mass and V_i is the effective potential given by the band-edge.

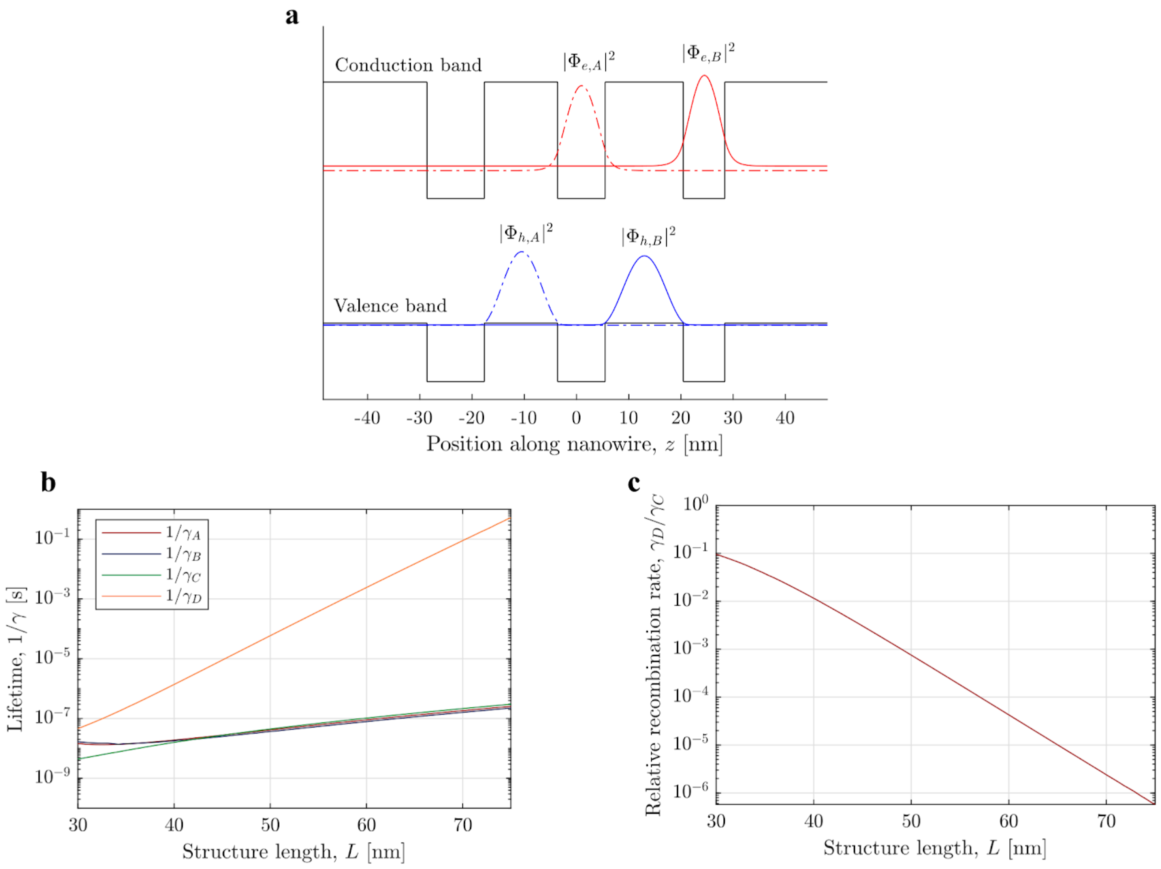

Figure S1 | Electron and hole wavefunctions and exciton lifetimes in a type-II QD array. **a,** Electron (red curves) and hole (blue curves) envelope wavefunction z-dependencies of a type-II multi-QD structure of size $[d_1, d_2, d_3, d_4, d_5] = [11nm, 14nm, 9nm, 15nm, 8nm]$. The wavefunctions are displaced vertically reflecting their energy levels. The distance between the valence and conduction band here is smaller than the actual energy gap, to enhance visibility. **b,** Exciton lifetimes, $1/\gamma$, as a function of structure length **c,** Recombination rate of X_D relative to the recombination rate of X_C, γ_D/γ_C, as a function of the structure length $L = d_1 + d_2 + d_3 + d_4 + d_5$, keeping the relative dimensions constant.

For a cylindrically symmetric nanowire, the single-particle wave function factorizes into a radial part and a z-directional part, $\psi_i(r) = \psi_i^R(R, \theta)\, \psi_i^z(z)$. Assuming an infinite potential outside the nanowire, the radial electron and hole wavefunctions will have a perfect overlap, so we only need to consider the z-directional wavefunctions, i.e. $|\langle\psi_h|\psi_e\rangle|^2 = \int dz \psi_h^z(z)^* \psi_e^z(z)$. The wavefunctions $\psi_e^z(z)$ are calculated numerically for QDs of various sizes in a nanowire of total length $140nm$. This value was chosen sufficiently large to avoid confinement effects due to the ends of the nanowire. The z-directional potential is illustrated in figure S1a for a particular multi-QD structure, along with the z-dependence of the relevant wavefunctions. Figure S1b plots the lifetimes of the excitons X_A, X_B, X_C, and X_D, for different structure lengths. This shows that the lifetimes of excitons X_A, X_B and X_C are of similar magnitude and much smaller than that of X_D. Figure S1c shows that the ratio γ_D/γ decreases exponentially with structure size.

Effective electron mass, m_e	$0.068m_0$
Effective hole mass, m_h	$0.64m_0$
Momentum matrix element $\lvert M\rvert^2$	$10.35\ eV \cdot m_0$
Conduction band depth, $V_{e,WZ} - V_{e,ZB}$	$129meV$
Valence band depth, $V_{h,ZB} - V_{h,WZ}$	$65meV$
ZB bandgap	$1.410\ eV$
Refractive index, n	3.44

Table S1 | InP band parameters[3–5]

S2. Effect of Coulomb interactions

The effect of Coulomb interactions is to hybridize different energy-levels, reducing the total energy of the system by bringing opposite charges closer, at the cost of occupying higher energy states[6]. Due to the large energy separation between the z-directional wavefunctions, the effect of Coulomb interactions will be negligible in the z-direction. However, for a typical nanowire diameter of d = 50nm the radial wavefunctions are closer spaced in energy and as a result, the radial wavefunctions can hybridize to enhance the electron/hole overlap, resulting in a larger dipole-moment and thus faster decay rate. This effect will be strongest for charges closer together, i.e. the effect is larger on excitons X_A, X_B, and X_C compared to X_D, and so our considerations without Coulomb interactions are overestimating the ratio γ_D/γ and therefore underestimating the maximum fidelity.

Coulomb interactions also modify the energy level and decay rate of one exciton depending on the presence of a neighbouring exciton. Thus the transition $|X_A X_B\rangle \to |X_B\rangle$ has a slightly different energy and decay rate compared to the transition $|X_A\rangle \to |g\rangle$. As a consequence of this, a single laser will be resonant on only one of these transitions.

S3. QD charging dynamics

The following Master Equation including spontaneous recombination is used to calculate the charging fidelity:

$$d\rho/dt = - i/\hbar[\hat{H}, \rho] + \sum_{i,j} \gamma_{i\to j} D(|j\rangle\langle i|)\rho + \sum_{i} \gamma_{dephasing} D(|i\rangle\langle i|)\rho \qquad (1)$$

Where ρ is the density matrix of the system, $|i\rangle \in \{|g\rangle, |X_A\rangle, |X_B\rangle, |X_A X_B\rangle, |X_D\rangle\}$ are the possible states (see Fig. 2b of the main text), $D(L)\rho = L\rho L^\dagger - (LL^\dagger\rho + \rho LL^\dagger)/2$ is the

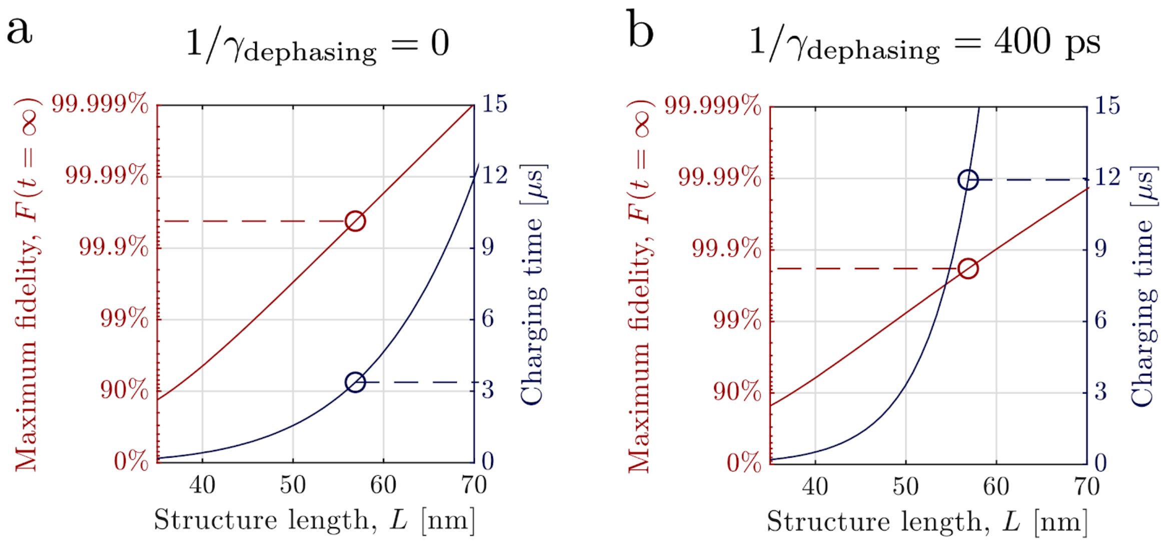

Figure S2 | Effect of pure dephasing. a, Maximum fidelity and corresponding charging time without pure dephasing (same as Fig. 3b of main text). **b,** Fidelity and charging time when including a pure dephasing time of 400ps.

Lindblad super-operator, $\gamma_{i \to j}$ is the spontaneous emission rate from state $|i\rangle$ to state $|j\rangle$ and $\gamma_{dephasing}$ is the pure dephasing rate. $\hat{H}$ is the Hamiltonian given by $\hat{H} = \hat{H}_0 + \hat{H}_{int}$, where $\hat{H}_0$ is the Hamiltonian of the unperturbed system, $\hat{H}_0 = \sum_i \hbar\omega_i |i\rangle\langle i|$, and $\hat{H}_{int}$ is the interaction Hamiltonian which we here approximate by

$$\hat{H}_{int} = \hbar\Omega_A cos(\omega_A t)(|g\rangle\langle X_A| + |X_A\rangle\langle g|) + \hbar\Omega_B cos(\omega_B t)(|X_A\rangle\langle X_A X_B| + |X_A X_B\rangle\langle X_A|)$$

This approximation is done by assuming that each laser only drives a single transition due to its detuning from the other transitions. The differences between the different transitions energies are on the order of 1 meV, while the Rabi frequencies of the lasers are on the order of a few μeV, justifying this approximation of the interaction Hamiltonian. Ω_A and Ω_B are the Rabi frequencies of the lasers A and B with frequencies ω_A and ω_B respectively. Here we consider only the case of equal laser driving strengths, $\Omega_A = \Omega_B \equiv \Omega$ for simplicity. The second term of equation (1) describes spontaneous recombination where the summation is

over the relevant spontaneous decay paths as shown by the dashed arrows of Fig. 2b in the main text. Assuming $\gamma \gg \gamma_D$ and, $\gamma \gg \gamma_{dephasing}$ and $\Omega \gg \gamma$ (see main text), the steady-state solution of equation (1) in the rotating wave approximation gives:

$$F(t=\infty) \equiv \langle X_D|\rho(t=\infty)|X_D\rangle = 1 - 4\gamma_D/\gamma$$

Although resonant manipulation of excitons in type-II QDs remains to be experimentally demonstrated, the regime $\Omega/\gamma \gg 1$ is feasible, as $\Omega/\gamma \propto 1/d$, where d is the dipole moment of the transition. In type-I QDs $\Omega/\gamma > 1$ is easily achieved[7] without any causing unwanted issues such as heating, and so it will also be achievable in type-II structures where the dipole moment is smaller due to the smaller electron-hole overlap.

Figure S2 shows the effect of pure dephasing. Adding dephasing does not significantly reduce the fidelity of the charging protocol, although it does increase the charging time compared to the case without dephasing. This is due to the less efficient pumping of the system when the excitons dephase.

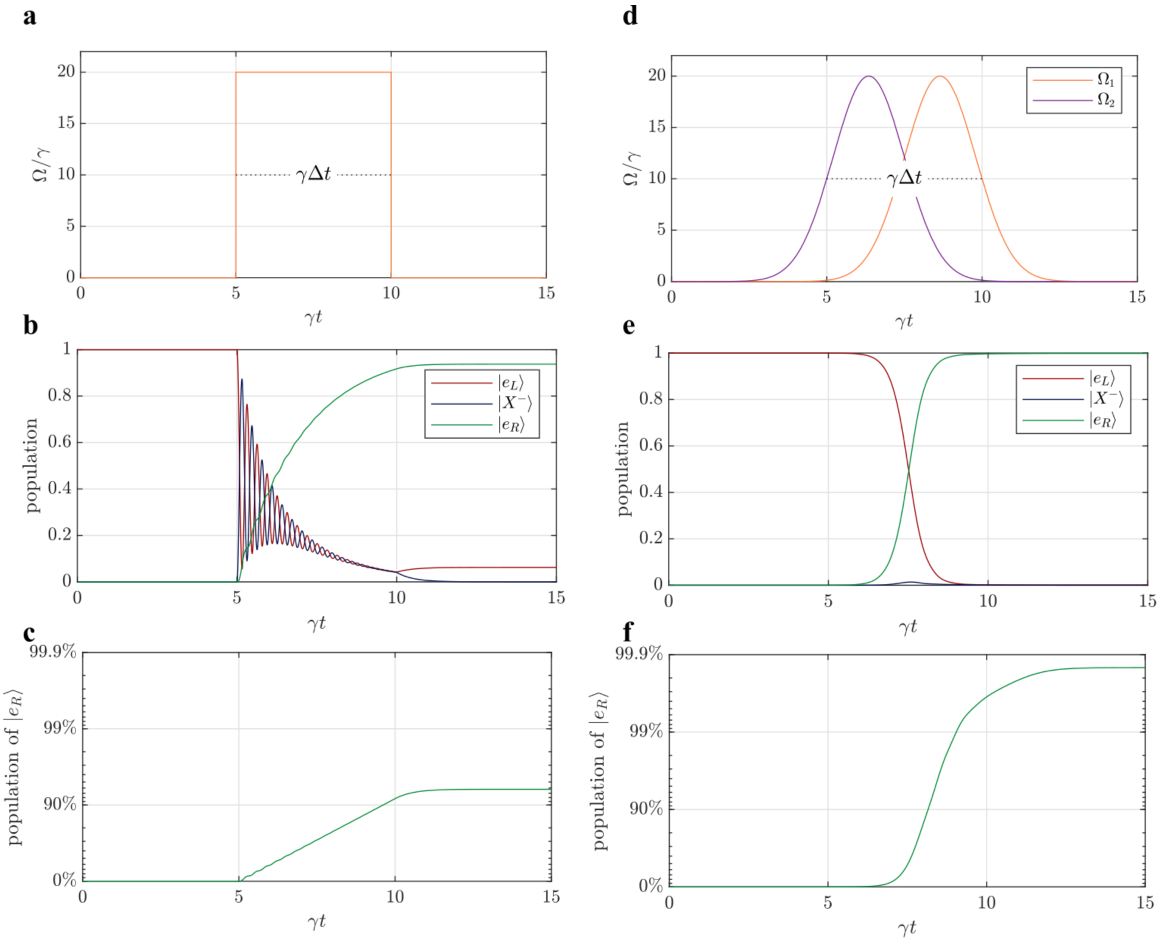

Figure S3 | Incoherent and coherent charge transfer. a, Pulse shape for incoherent charge transfer. **b,** Populations of the states $|e_L\rangle$, $|X^-\rangle$ and $|e_R\rangle$ of the Λ-system (fig. 4a in the main text) under incoherent charge transfer using a single laser. **c,** Population of the target state $|e_R\rangle$. **d-f,** same as **a-c**, but for coherent charge transfer using the STIRAP scheme. The transfer duration here is $T_0 = 5\gamma$ and the peak Rabi frequencies are $\Omega = 20\gamma$.

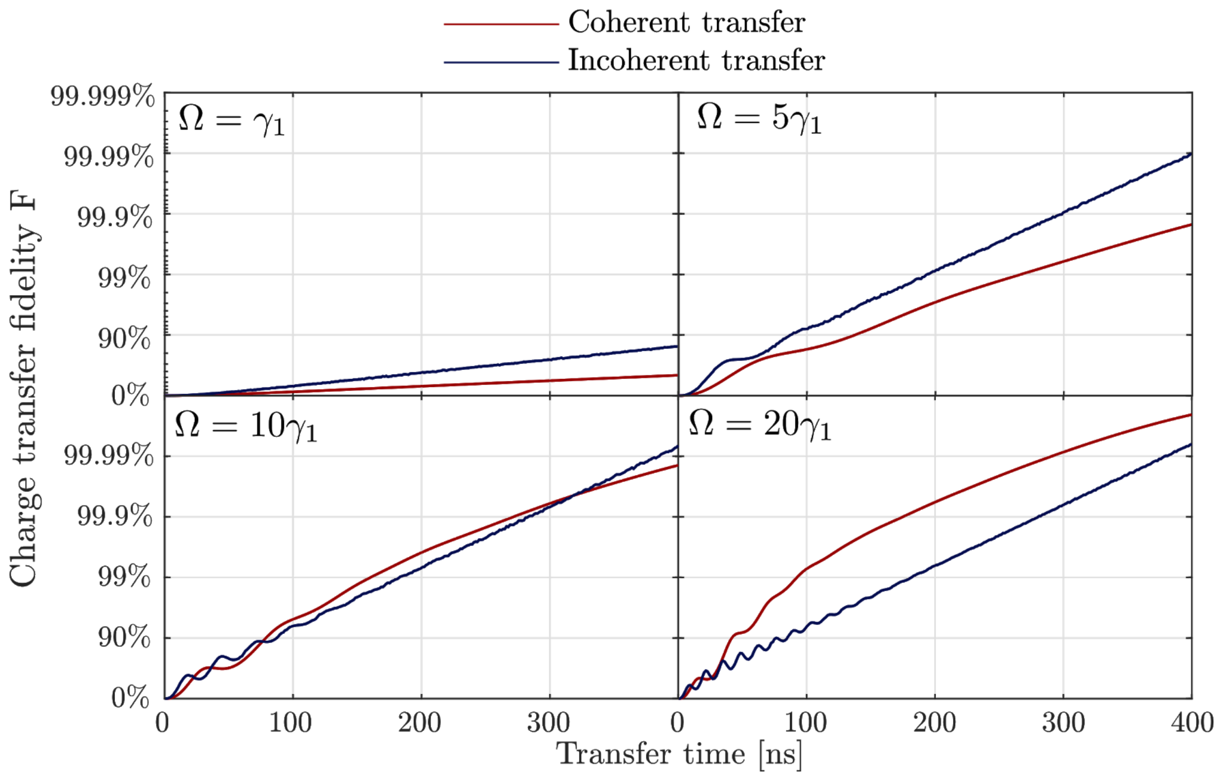

Figure S4 | Charge transfer fidelity dependence on pulse intensities.

S4. Coherent and incoherent charge transfer dynamics

The charge transfer scheme is modelled similarly to the charging scheme i.e. using a Master equation of the Λ-system of Fig. 4a of the main text. For incoherent transfer we model a single driving laser as a square pulse (Fig. S3a):

$$\Omega_1(0) = \Omega_0[\Theta(t - t_0) - \Theta(t - t_0 - \Delta t)], \qquad \Omega_2(t) = 0$$

Where $\Theta(t)$ is the Heaviside step function and Ω_0 is the peak Rabi frequency. Ω_1 drives population from $|e_L\rangle$ to $|X^-\rangle$, while spontaneous decay of the left electron with the hole transfers population from $|X^-\rangle$ to $|e_R\rangle$. The temporal evolutions of the three states are shown in Fig. S3b, while the population of only the target state $|e_R\rangle$ is highlighted in Fig. S3c. For coherent transfer we consider the stimulated Raman adiabatic passage (STIRAP) scheme[8,9], which allows for a high fidelity population transfer with large stability against

experimental imperfections, such as, for example, fluctuations of the laser parameters. The STIRAP scheme drives the two transitions with partially overlapping Gaussian pulses (Fig. S3d):

$$\Omega_1(t) = \Omega_0 exp(-(t - t_1)^2/(2\tau^2)), \qquad \Omega_2(t) = \Omega_0 exp(-(t - t_2)^2/(2\tau^2))$$

where t_1 and t_2 are the center of the pulses and τ is their widths. This allows a coherent transfer of population directly from $|e_L\rangle$ to $|e_R\rangle$, preserving any quantum correlations of the electron during the transfer. During the transfer, the intermediate occupation of the trion state, $|X^-\rangle$, is greatly suppressed as shown in Fig. S3e, resulting in suppressed decoherence due to the spontaneous decay of $|X^-\rangle$. The pulse delay is set to $t_1 - t_2 = -2\tau$. Numerical optimization of this delay depending on the pulse width, τ , and peak Rabi frequency Ω_0 would allow for even higher transfer fidelities. We quantize the duration of these processes in terms of the full width at half maximum, Δt, of the combined pulse sequence, as indicated in Fig. S3d.

Fig S4 shows how the charge transfer fidelity depends on the pulse intensities. The fidelity of the incoherent transfer process is mostly independent of pulse intensity when $\Omega \gg \gamma_1$, as it is limited by the recombination rate γ_2 , independent of Ω . The coherent STIRAP process on the other hand benefits from higher pulse intensities, as a high pulse intensity better suppresses the occupation of the intermediate trion state, $|X^-\rangle$, and thus reduces the effect of unwanted decay back into the initial state $|e_L\rangle$.

S5. Proposal for experimental verification of long-lived charges

Here, we consider the first steps towards a feasible experimental implementation of the charge generation protocol. That is, we propose how to generate a spatially separated

electron-hole pair—the long-lived exciton, X_D—and verify its presence. We ignore pure dephasing in the following for simplicity, but the method outlined works equally well when pure dephasing is present. We require two lasers, A and B, both of which should be on resonance with their respective transitions, $|g\rangle \rightarrow |X_A\rangle$ and $|X_A\rangle \rightarrow |X_A X_B\rangle$, to maximize the fidelity of the scheme. Figure S5a shows the charging fidelity at steady state as a function of laser detuning from their respective resonances, denoted Δ_A and Δ_B. Scanning the lasers near the resonance frequencies reveals two traits of the system: First, we observe a prominent diagonal line in Fig. S5a showing efficient pumping when $\Delta_A + \Delta_B = 0$. This corresponds to a two-photon excitation, i.e. the total frequencies of the lasers add up to effectively pump population directly from the ground-state $|g\rangle$ to $|X_A X_B\rangle$, from which it can decay into state $|X_D\rangle$. Note that this is a two-laser two-photon excitation process, and should not be confused with single-laser two-photon processes, e.g. as typically used for coherent bi-exciton excitation in QDs[10,11]. Secondly, we observe a vertical line, due to partial excitation of both X_A and X_B whenever laser A on resonance, even if laser B is detuned. This is a result of the long lifetime of X_D: even weak excitation of state $|X_A X_B\rangle$ from a detuned laser B eventually leaves the system in state $|X_D\rangle$. In the opposite case, i.e. when laser B is on resonance while laser A is detuned, the excitation of both X_A and X_B is much less efficient, as seen by the lack of a horizontal line. This asymmetry originates from the increased dephasing of transition $|X_A\rangle \leftrightarrow |X_A X_B\rangle$ compared to $|g\rangle \leftrightarrow |X_A\rangle$ due to the increased number of decays paths from state $|X_A X_B\rangle$. As a result, the transition $|X_A\rangle \leftrightarrow |X_A X_B\rangle$ is spectrally broader, meaning it can be pumped more efficiently with a detuned laser, compared to transition $|g\rangle \leftrightarrow |X_A\rangle$.

However, measuring the population of state $|X_D\rangle$ directly is not practical, due to the long lifetime of X_D, and so experimentally verifying the success of the charging scheme and determining the optimal laser tunings is not straightforward. Instead, the detection of the spontaneous recombination of X_A can serve as an efficient measure to verify the generation of X_D. The intensity of this fluorescence is proportional to the population of state $|X_A\rangle$ and is plotted in Fig S5b. We show that state $|X_A\rangle$ is populated whenever the laser B is detuned while laser A is on resonance, as expected.

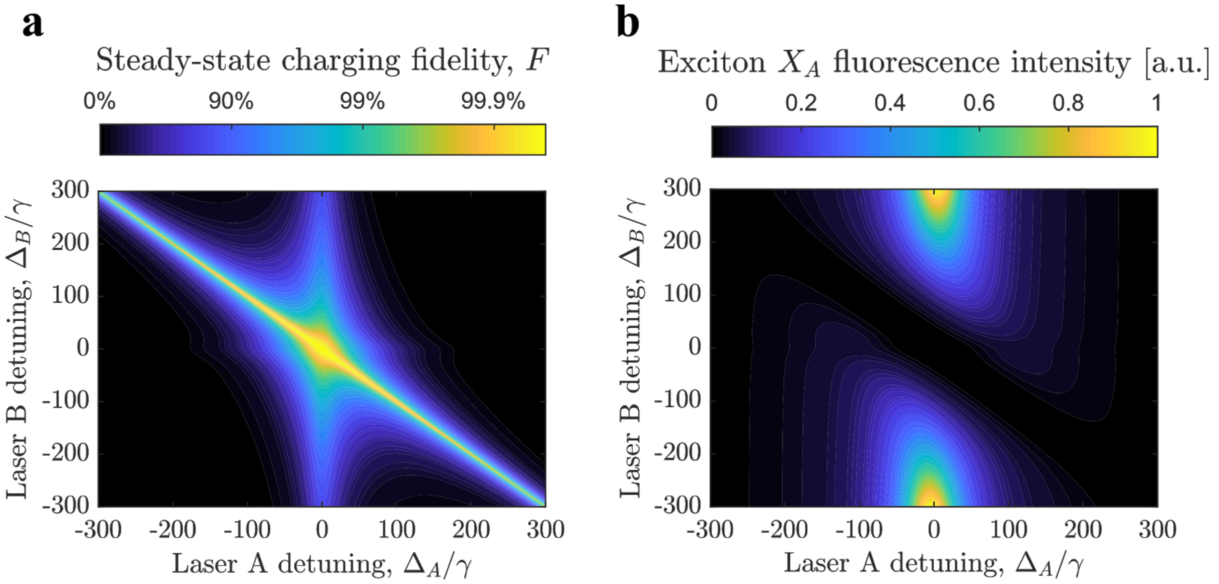

Figure S5 | Laser tuning dependence of the charge generation scheme. a, Steady-state charging fidelity. When both lasers are on resonance (the center of the plot) the fidelity is maximized, as expected. When both lasers are detuned such that their frequencies add up to the total energy difference from the ground state $|g\rangle$ to $|X_A X_B\rangle$, i.e. $\Delta_A + \Delta_B = 0$, efficient transfer still occurs as seen by the diagonal signature. **b**, Resonance fluorescence intensity from the spontaneous decay of X_A at steady-state. When both lasers are on resonance the system at steady-state is trapped in state $|X_D\rangle$ and the fluorescence is therefore quenched. When only laser A is on resonance, exciton X_A is populated, and radiates due to spontaneous decay. The diagonal two-photon excitation line is seen as an imprint in the fluorescence intensity. Parameters in both figures are $\gamma_D/\gamma = 10^{-4}$ and $\Omega_A = \Omega_B = 20\gamma$.

When both lasers are on resonance the fluorescence is quenched as the population is transferred to $|X_A X_B\rangle$ and subsequently $|X_D\rangle$, corresponding to the desired charging. Of course, $|X_A X_B\rangle$ can also decay to states $|X_A\rangle$ or $|X_B\rangle$ and then $|g\rangle$, but these states will be quickly be pumped back to $|X_A X_B\rangle$ until a decay to $|X_D\rangle$ occurs, eventually trapping the system in state $|X_D\rangle$. The quenched fluorescence of $|X_A\rangle$ therefore indicates the generation of X_D. Furthermore, we can see an imprint of the diagonal two-photon excitation line from Fig. S5a in Fig. S5b. Experimental characterisation of our model can thus be obtained in this resonance fluorescence measurement.

References

1. Carmichael, H. J. *Statistical Methods in Quantum Optics 1: Master Equations and Fokker-Planck Equations*. (Springer Science & Business Media, 2013).
2. Coldren, L. A., Corzine, S. W. & Mashanovitch, M. L. *Diode Lasers and Photonic Integrated Circuits*. (John Wiley & Sons, 2012).
3. Adachi, S. Optical dispersion relations for GaP, GaAs, GaSb, InP, InAs, InSb, AlxGa1−xAs, and In1−xGaxAsyP1−y. *J. Appl. Phys.* **66**, 6030–6040 (1989).
4. Vurgaftman, I., Meyer, J. R. & Ram-Mohan, L. R. Band parameters for III–V compound semiconductors and their alloys. *J. Appl. Phys.* **89**, 5815–5875 (2001).
5. Bouwes Bavinck, M. *et al.* Photon Cascade from a Single Crystal Phase Nanowire Quantum Dot. *Nano Lett.* **16**, 1081–1085 (2016).
6. Taherkhani, M., Willatzen, M., Mørk, J., Gregersen, N. & McCutcheon, D. P. S. Type-II quantum-dot-in-nanowire structures with large oscillator strength for optical quantum gate applications. *Phys. Rev. B Condens. Matter* **96**, 125408 (2017).
7. Nick Vamivakas, A., Zhao, Y., Lu, C.-Y. & Atatüre, M. Spin-resolved quantum-dot resonance fluorescence. *Nat. Phys.* **5**, 198 (2009).
8. Bergmann, K., Theuer, H. & Shore, B. W. Coherent population transfer among quantum states of atoms and molecules. *Rev. Mod. Phys.* **70**, 1003–1025 (1998).
9. Taherkhani, M., Willatzen, M., Denning, E. V., Protsenko, I. E. & Gregersen, N. High-fidelity optical quantum gates based on type-II double quantum dots in a nanowire. *Phys. Rev. B Condens. Matter* **99**, 165305 (2019).
10. Brunner, K., Abstreiter, G., Böhm, G., Tränkle, G. & Weimann, G. Sharp-line photoluminescence and two-photon absorption of zero-dimensional biexcitons in a GaAs/AlGaAs structure. *Phys. Rev. Lett.* **73**, 1138–1141 (1994).
11. Stufler, S. *et al.* Two-photon Rabi oscillations in a single In_xGa_xAsGaAs quantum dot. *Phys. Rev. B Condens. Matter* **73**, 125304 (2006).
